# The potential of stem cells in breast cancer therapy: A review

**DOI:** 10.1097/MD.0000000000048829

**Published:** 2026-05-15

**Authors:** Zundong Liu, Dachun Wang

**Affiliations:** a Stem Cell Center, Second Affiliated Hospital of Fujian Medical University, Quanzhou, Fujian Province, China; b The Brown Foundation Institute of Molecular Medicine for the Prevention of Human Diseases, University of Texas Medical School at Houston, Houston, TX.

**Keywords:** breast cancer strategy, MSC-derived exosomes, MSCs, stem cells, underlying mechanisms

## Abstract

Breast cancer remains the most prevalent malignancy among women worldwide, underscoring the urgent need for innovative therapeutic strategies. Stem cell–based anticancer approaches have attracted substantial attention because of their ability to generate immune-related cells and selectively home to tumor sites, among which mesenchymal stem cells (MSCs) are considered particularly promising owing to their strong tumor‐tropic capacity and low immunogenicity. In this review, current evidence on the biological functions and therapeutic potential of MSCs and MSC-derived exosomes in breast cancer was systematically compiled and analyzed, with particular emphasis on MSC-mediated cytokine signaling, modulation of the tumor microenvironment, and the molecular cargo and functional effects of MSC-derived exosomes. The available data indicate that MSCs exert context-dependent dual effects, either promoting or inhibiting tumor growth through secretion of cytokines and activation of intercellular signaling pathways, while exosomes derived from MSCs likewise display both antitumor and protumor activities yet retain intrinsic tumor-targeting capability. Notably, compared with live MSCs, exosomes demonstrate greater stability, reduced immunogenic risk, and enhanced feasibility as therapeutic delivery vehicles, highlighting their translational potential. Accumulating evidence suggests that immune regulation and remodeling of the tumor microenvironment represent central mechanisms underlying these bidirectional effects. Collectively, MSCs and their derived exosomes constitute promising therapeutic platforms for breast cancer; however, their context-dependent regulatory properties necessitate rigorous mechanistic investigation and careful optimization prior to clinical application, and a deeper understanding of the molecular pathways through which they influence tumor progression will be essential for the development of safer and more effective stem cell–based anticancer therapies.

## 1. Introduction

Breast carcinoma is one of the most prevalent cancers in women, and despite the availability of a variety of drugs and treatment options to treat breast cancer, it still has the second highest mortality rate.^[1,2]^ Ordinary radiotherapy drugs have the same strong killing effect on nontumor tissues that limit the dose that can be used, thus leading to the inability to completely eradicate tumors.^[3,4]^ Another treatment strategy is to deliver the drug at the tumor site to reduce off-target effects. This requires a carrier that is highly selective concerning the tumor site, and stem cells and exosomes from stem cells are attractive candidates for such therapy.^[5–8]^

Stem cells were first discovered by Mcculloch, a group of cells that are not fully differentiated and have the ability to regenerate various tissues and organs.^[9,10]^ Stem cells include embryonic stem cells (ESCs), adult stem cells, and induced pluripotent stem cells (iPSCs). Adult stem cells from various organs include neural stem cells (NSCs), hematopoietic stem cells, epidermal stem cells (ESCs), and mesenchymal stem cells (MSCs).^[11–13]^ MSCs are extensively researched in clinical tissue engineering due to their wide variety of anti-inflammatory and immunomodulatory capabilities, ease of expansion under culture conditions, and capacity to differentiate and create a large number of beneficial cytokines.^[14]^ MSCs were initially found in bone marrow (bone marrow mesenchymal stem cells, BMSCs); since then, MSC-like populations have been isolated from various tissues and organs, including adipose tissue (adipose-derived stem cells, ADSCs),^[15]^ human umbilical cord tissue (human umbilical cord mesenchymal stem cells, HUMSCs),^[16]^ peripheral blood (peripheral blood-derived mesenchymal stem cells, PBMSCs)^[17]^ and dental pulp (dental pulp stem cells, DPSCs).^[18]^ Comprehensive studies of the transcriptome of MSCs have shown that MSCs of different tissue origins have heterogeneous markers.^[19]^ In an ideal organism, stem cells can move to several different tissues and organs. However, stem cells go to the injured location preferentially when there is damage there.^[20]^ Tumor environment-associated cells and tumor cells are capable of producing high local concentrations of cytokines, mimicking the environment of tissue injury.^[21]^ Although stem cell-based approaches have demonstrated significant therapeutic promise in several illnesses, their application to cancer treatment is still debatable. While some research suggests that stem cells may promote cancer development, other evidence point to the positive impacts of stem cells in tumor treatment.^[22]^ In this review, we provide an overview of the functions of stem cells, MSCs, and MSC-derived exosomes in breast cancer and discuss the dual roles of stem cells in cancer treatment.

## 2. The potential use of ESCs and iPSCs in breast cancer treatment

ESCs have the ability of unlimited self-renewal, lack contact inhibition, and form teratomas in immunodeficient mice.^[23]^ Some research has shown that conditioned medium (CM) from ESCs can inhibit the proliferation, invasion, and stemness and promote apoptosis of MDA-MB-231 cells. Further studies found that ESC-CM decreased the expression of epithelial − mesenchymal transition (EMT)-related proteins such as fibronectin, vimentin, and snail.^[24]^ Mouse ESC-CM suppressed 4T1 cell growth, metastasis, and angiogenesis by inhibiting Stat3.^[25]^ Immunization of mice with ESC-exosomes expressing GM-CSF as cancer prevention was extremely efficient in blocking the implantation of lung cancers and no toxicity.^[26]^ Therefore, exosomes from ESCs carrying GM-CSF may prevent breast cancer. However, further studies to define the key anticancer factors expressed in the exosomes as well as in ESC-CM should be necessary to be carried out. More clarification of the kinds of cytokines in ESC-CM that function during targeted breast cancer therapy is needed, which could open a novel targeting therapy in breast cancer treatment. The use of ESCs in therapy is limited due to the potential of teratoma formation, as such, ESC transformation into various cell types for tumor treatment is drawing greater attention. Some studies have attempted to induce the conversion of ESCs into endothelial cells (ESC-ECs), these cells inhibited the proliferation and migration of MDA-MB-231 cells by suppressing the Wnt signaling pathway, in vivo experiments showed that intravenous injection of ESC-ECs bearing suicide genes further inhibited the proliferation of MDA-MB-231 cells in tumors.^[27]^ To further develop novel therapeutic approaches for treating breast cancer, it is necessary to identify the specific components in ESC-ECs that prevent cancer. Although ESC-derived differentiated cells may provide an almost limitless source of cells for anticancer therapy, the presence of undifferentiated ESCs in differentiated cell populations may cause the formation of teratomas. Therefore, the preparation of high-purity “carriers” is a major priority during the development of ESC-mediated tumor therapies. Regarding therapeutic applications, one concern for ESCs is immunological rejection following transplantation. To overcome this issue, increasing attention is being focused on iPSCs derived from donor somatic cells.^[28]^ Some research has shown that MSCs with unlimited differentiation potential and uniform homing ability can be obtained from iPSCs, and doxorubicin-loaded iPSC-MSCs and their exosomes had strong tumor targeting, reduced TNBC tumor metastasis and decreased cancer incidence and tumor burden in mouse models.^[29]^ The similar transcriptome and antigens of iPSCs and tumor cells make iPSCs an effective source of antigens for tumor vaccine production. Studies have demonstrated that preventative iPSC-based vaccines can inhibit tumor growth in mice with breast cancer, mesothelioma, and melanoma, prevent recurrence of resected melanoma, and reduce metastasis.^[30,31]^ A subsequent study found that iPSC-derived vaccines were similarly efficacious in pancreatic cancer treatment. iPSC vaccines significantly inhibit pancreatic cancer cell proliferation. Regarding immune effects, the vaccines increase the levels of CD8 + cytotoxic T cells and decrease the levels of tumor-promoting immune cells such as T helper 17 cells.^[32]^ In summary, the main focus of iPSC-derived therapy is to activate immune-related cells against the tumor. Studies have shown that iPSCs can be differentiated into natural killer (NK) cells, T cells, etc, for tumor therapy.^[33–35]^ Although iPSCs can provide abundant cell numbers for the generation of immunotherapies, the tumorigenic potential of iPSCs is an additional factor limiting their application. In the field of anticancer research, ESCs and iPSCs can be used to generate antitumor cells and vectors, therefore currently research on stem cell therapies for tumor treatment research is mostly focused on MSCs. Table 1 provides an overview of the research covered in this section.

**Table 1:** The utility of ESCs and iPSCs in breast cancer treatment.

Stem cell type	Method	Breast cancer model	Key cargo	Effect	Proposed mechanism	References
ESC-CM	In vitro	MDA-MB- 231 cells	N/A	Proliferation↓ Invasion↓ Stemness↓ Apoptosis↑	Fibronectin↓ Vimentin↓ Snail↓	24
Mouse ESC-CM	In vitro and in vivo	4T1 cells	N/A	Growth↓ Metastasis↓ Angiogenesis↓	Stat3 signaling↓	25
ESC-derived exosomes	In vivo	B16-F10, MC-38, and E0771 cells	GM-CSF	Proliferation↓	CD8 + T-cell number↑	26
ESC- ECs	In vitro and in vivo	MDA-MB- 231 cells	Suicide genes	Proliferation↓ Migration↓	Wnt signaling↓	29
iPSC vaccine	In vitro and in vivo	pancreatic cancer	N/A	Proliferation↓	CD8 + T-cell number↑ T helper 17 cell ↑	32

CM = conditioned medium, ESC-ECs = ESC-derived endothelial cells, ESCs = embryonic stem cells, iPSC-EPCs = iPSC-derived endothelial progenitor cells, iPSC-MSCs = iPSC-derived MSCs, iPSCs = induced pluripotent stem cells.

## 3. MSC utility in preventing breast cancer development

Previous studies have demonstrated that ADSCs with low CD90 expression generated by LPS induction and exosomes derived from these cells can significantly inhibit tumor growth, reduce the proliferation and migration of tumor cells and enhance apoptosis, CD90^low^ exosomes loaded with miRNA-16-5p further increased anticancer activity.^[36]^ Some research has indicated that CM from MSCs reduced Stat3 activity and improved the chemotherapy sensitivity of breast cancer cells in vitro and vivo. Furthermore, the CM could decrease the ALDH-positive cancer stem cell (CSC) population and reduce cell migration.^[37]^ MSC-CM inhibited breast cancer cell growth by downregulating β-catenin in MCF-7 cells. Further studies revealed that this effect was associated with secreted Dickkopf-1 (DKK-1) in MSC cells.^[38]^ The extracellular matrix of adult stem cells produced by HUMSCs increases PTEN protein expression in tumor cells and inhibits MDA-MB-231 cell growth by inducing the secretion of DKK-1.^[39]^ Similarly, CM from ADSCs inhibits cell viability and reduces mitochondrial membrane potential as well as the migration capacity of MCF-7 and MDA-MB-231 cells. Further research has shown that ADSC-CM decreased the expression of β-catenin, cyclin D1 and Bcl-xL protein.^[40]^ The above findings suggest that CM from MSCs may contain secretory factors that have an inhibitory effect on tumor cells. Consequently, identifying the important MSC secretory components is crucial.^[41]^ During tumor therapy, MSCs could inhibit cell invasion and metastasis of T47D and MDA-MB-231 cells by secreting TIMP in a coculture environment.^[42]^ MDA-MB-231 cells undergo apoptosis when co-cultured with HUMSCs, and HUMSCs inhibit the growth of in situ and metastatic breast cancer tumors in mouse xenografts.^[43]^ Low FST expression is associated with poor prognosis in patients with TNBC.^[44]^ Some studies have revealed that FST is highlighted enriched in HUMSCs, thus, HUMSCs may reduce tumor burden through FST-related signaling pathways in mouse mammary lung metastasis models.^[45]^ When MCF-7 cells were treated with HUMSC-CM alone or in combination with atorvastatin, the capacity of cells to migrate and proliferate was suppressed.^[46,47]^ In a dilated cardiomyopathy rat model, HUMSCs alleviated interstitial fibrosis and cardiac dysfunction by effectively inhibiting the TGF-β1/ERK1/2 signaling pathway.^[48]^ It could be intriguing to examine the potential of HUMSC-based modulation of the TGF-β signaling pathway in the treatment of breast cancer. Human endometrial mesenchymal stem cells (EnSCs), derived from menstrual blood may be a new source of adult mesenchymal stem cells. The antitumor effects of EnSCs in epithelial ovarian cancer include causing cell cycle arrest, boosting apoptosis, dysregulating mitochondria, decreasing pAKT, and enhancing FoxO3a nuclear translocation.^[49]^ Human amniotic fluid mesenchymal stem cells (hAFMSCs) can be obtained from amniocentesis in the middle or end of pregnancy.^[50]^ Ovarian cancer SKOV3 cell activity is effectively suppressed by hAFMSCs-released cytokines.^[51]^ IFNα-AFMSCs significantly suppressed tumor growth, inhibited angiogenesis, and promoted apoptosis in cervical cancer.^[52]^ Human uterine cervical stem cells (hUCESCs) were isolated from the normal human uterine cervix and had shown antitumor effects against HeLa, MDA-MB-231 cells.^[53]^ The unique antitumor characteristics of MSCs derived from cells of reproductive tissues provide natural defense mechanisms to prevent the migration of cancer cells from the mother to the fetus, which explains why tumors are rare in fetuses.^[54]^ The antitumor activity of such cells may make them a novel tool for targeted breast cancer therapy in the future.

The tumor-homing properties of MSCs and AFMSCs make them promising carriers for the precise and selective delivery of anticancer proteins and drugs.^[55]^ Stueny was the first to employ MSCs as a carrier for antitumor treatment. In 2002 and 2004, MSCs were used to deliver IFN-β for antitumor therapy.^[56,57]^ Similarly, BMSCs bearing IFN-β have been reported to significantly reduce the growth of metastatic human breast cancer in the lungs of SCID mice when administered in combination with 5-fluorouracil.^[58]^ Some studies showed that overexpression of IL-12 in mouse MSCs significantly interfered with the growth of 4T1 cells in vivo through immunomodulation, and IL-12-overexpressing MSCs cells embedded in stroma have stronger antitumor effects.^[59]^ Even though there have not been many recent studies, the above studies suggest that strategies that allow the transfer of tumor immunotherapy-related variables to MSCs may be a beneficial research direction. Azurin is a bacterial protein that has shown anticancer activity in a variety of cancer models both in vitro and in vivo.^[60–62]^ Related research studies have found that CM from MSCs overexpressing azurin increases death and inhibits the proliferation, migration, and invasive ability of MCF-7 breast cancer cells.^[63]^ TNF-related apoptosis-inducing ligand (Trail) induces apoptosis in a wide variety of cancer models,^[64–66]^ however, challenges to the intravenous administration of Trail, including its short pharmacokinetic half-life.^[67]^ MSCs expressing Trail decreased a variety of types of cancer cell viability in vitro under coculture conditions. In a breast cancer pulmonary metastasis model, 38% of metastatic disease was completely cleared.^[68]^ These results suggest that such therapies can be used for a wide range of indications, including to treat primary tumors and their metastases. Recently, some research has demonstrated that MSCs overexpressing Lrp5, β-catenin, Snail, or Akt have tumor-suppressing capabilities, and administration of these cells reduced the development of mammary tumors and tumor-driven bone loss.^[69]^ Tumor cell lysis occurs when tumor cells are exposed to conditionally replicating adenoviruses (CRAds).^[70]^ MSC-beared CRAds effectively inhibited breast cancer-derived pulmonary metastases and prolonged overall survival in a xenograft model.^[71]^ In terms of the targeted delivery of antitumor drugs, both DPSCs, and ADSCs are capable of encapsulating paclitaxel and inhibiting the growth of breast cancer cells, and DPSCs are better tolerated than BMSCs as transporters of paclitaxel.^[72]^ ADSCs can be used during breast reconstruction to locally deliver additional chemotherapeutic drugs to prevent tumor recurrence, and the anticancer action of PTX-loaded ADSCs may make them a novel tool.^[73]^ 5-Fluorocytosine (5-FC) has low toxicity as a precursor drug for 5-fluorouracil (5-FU). Administration of MSCs expressing cytosine deaminase to specifically catalyze the conversion of 5-FC into 5-FU at the tumor site is effective in the treatment of glioblastoma multiforme, and the strategy-induced cell cycle arrest and DNA damage in vitro and inhibited the growth of tumors in vivo.^[74]^ The prodrug catalyticase can improve tumor cell-specific killing by being transported by MSCs, catalyzing the conversion of low-toxicity prodrugs into drugs with tumor cell-killing properties at the tumor site and reducing the systemic toxicity of drugs against organ tissues. The limited scalability and donor variability of tissue-derived MSCs hinder their application, but MSCs produced from iPSCs can provide near-limitless therapeutic materials.^[29]^ MSCs produced from iPSCs, on the other hand, guarantee a single source and prevent treatment variability brought on by several sources. Our lab has developed a quite comprehensive approach to produce a pure population of ESC- and iPSC-derived lung tissue progenitor, which could be used to separate MSCs with high purity, providing the groundwork for improving the efficacy of clinical therapy.^[75–77]^ MSCs may also be useful for breast cancer imaging due to their ability to traffic toward tumor cell sites in vivo. In mice injected with the sodium iodide symporter-overexpressing BMSCs started to accumulate in the lung, heart, intestine, and tumor sites. However, after 7 days, the cells were depleted from nontumor tissues but remained in tumor tissues, providing a basis for radioiodine (^131^I)-based targeted therapy and increasing apoptosis of tumor cells. There were no ^131^I-related adverse effects during the 8 weeks of administration.^[78]^ Micro-vesicles from MSCs overexpressing miR-34a significantly inhibited migration and invasion and increased the apoptosis of cancer cells.^[79]^ The studies mentioned in this section are summarized in Table 2.

**Table 2:** MSCs in breast cancer therapy.

MSC type	Method	Cell types	Key cargo	Effect	Proposed mechanism	References
CD90^low^ ADSCs	In vitro and in vivo	E0771 and 4T1 cells	MiRNA-16-5p	Tumor growth↓ Proliferation↓ Apoptosis↑	N/A	36
MSC-CM	In vitro and in vivo	MDA-MB- 231 cells	N/A	Chemotherapy sensitivity↑ ALDH^+^ CSC population↓ Cell migration↓	Stat3 signaling↓	37
MSC- CM	In vitro	MCF-7 cells	N/A	Cell growth↓	β-catenin↓ DKK-1↑	38
HUMSC-CM	In vitro	MDA-MB- 231 cells	N/A	Proliferation↓	DKK-1↑ PTEN↑	39
ADSC-CM	In vitro	MCF-7 and MDA-MB- 231 cells	N/A	Cell viability↓ Mitochondrial membrane potential↓ Migration↓	β-catenin↓Cyclin D1↓ Bcl-xL↓	40
MSCs	In vitro	T47D and MDA-MB- 231 cells	N/A	Metastasis↓ Invasion↓	TIMP↑ MMPs↓	42
HUMSCs	In vitro and in vivo	MDA-MB- 231 cells	N/A	Apoptosis↑ Metastasis↓	Ν/Α	43
HUMSCs	In vitro and in vivo	MDA-MB- 231 cells	FST	Cell growth↓ Tumor burden↓	Ν/Α	44
HUMSCs	In vitro	MCF-7 cells	N/A	Cell growth↓ Migration↓	Ν/Α	46,47
EnSCs	In vitro and in vivo	SKOV3 cells	N/A	Cell cycle arrest Apoptosis↑	pAKT↓ FoxO3a nuclear localization↑	49
AFMSC-CM	In vitro	SKOV3 cells	N/A	Cell viability↓	Ν/Α	51
AFMSCs	In vitro	HeLa cells	N/A	Angiogenesis↓ Proliferation↓ Apoptosis↑	Ν/Α	52
hUCESCs	In vitro	MDA-MB- 231 and HeLa cells	IFN-α	Proliferation↓Cell cycle arrest Apoptosis↑ Invasion↓	Ν/Α	53
MSCs	In vivo	MDA-MB- 231 and A375SM cells	IFN-β	Proliferation↓ Migration↓	Ν/Α	56,57
BMSC	In vivo	MDA-MB- 231 cells	IFN-β	Proliferation↓	Ν/Α	58
MSCs	In vivo	4T1 and B16 cells	IL-12	Proliferation↓ Angiogenesis↓	Ν/Α	59
HUMSCs/BMSCs	In vitro	MCF-7 and A549 cells	Azurin	Proliferation↓ Migration↓ Invasion↓ Apoptosis↑	Ν/Α	63
MSCs	In vivo	MDA-MB- 231, A549, H357, and HeLa cells	Trail	Migration↓	Ν/Α	68
MSC-CM	In vitro and in vivo	EO771, 4T1, MDA-MB- 231, RAW264.7, TRAMP-C2, and PC-3 cells	Lrp5, β-catenin, Snail, and Akt	Proliferation↓ Tumor-induced osteolysis↓	CXCL2↓ LIF↓ PDL1↓	69
HUMSCs	In vivo	MDA-MB- 231 cells	CRAds	Migration↓ Overall survival↑	Ν/Α	71
DPSCs/ADSCs	In vitro	MCF-7 cells	PTX	Cell growth↓	Ν/Α	72,73
MSCs	In vitro and in vivo	U87 cells	5-FC	Cell cycle arrest DNA damage Cell growth↓	Ν/Α	74
BMSCs	In vivo	MDA-MB- 231 cells	NIS	Proliferation↓	Ν/Α	78
ADSCs	In vitro	MDA-MB- 231 cells	MiR-34a	Migration↓ Invasion↓ Apoptosis↑	PD-L1↓	79

ADSCs = adipose-derived adult stem cells, BMSCs = bone marrow mesenchymal stem cells, CM = conditioned medium, DPSCs = dental pulp stem cells, EnSCs = human endometrial mesenchymal stem cells, hAFMSCs = human amniotic fluid mesenchymal stem cells, hUCESCs = human uterine cervical stem cells, HUMSCs = human umbilical cord mesenchymal stem cells, MSCs = mesenchymal stem cells.

## 4. MSC-derived exosomes suppress breast tumor development

**Figure 1.**
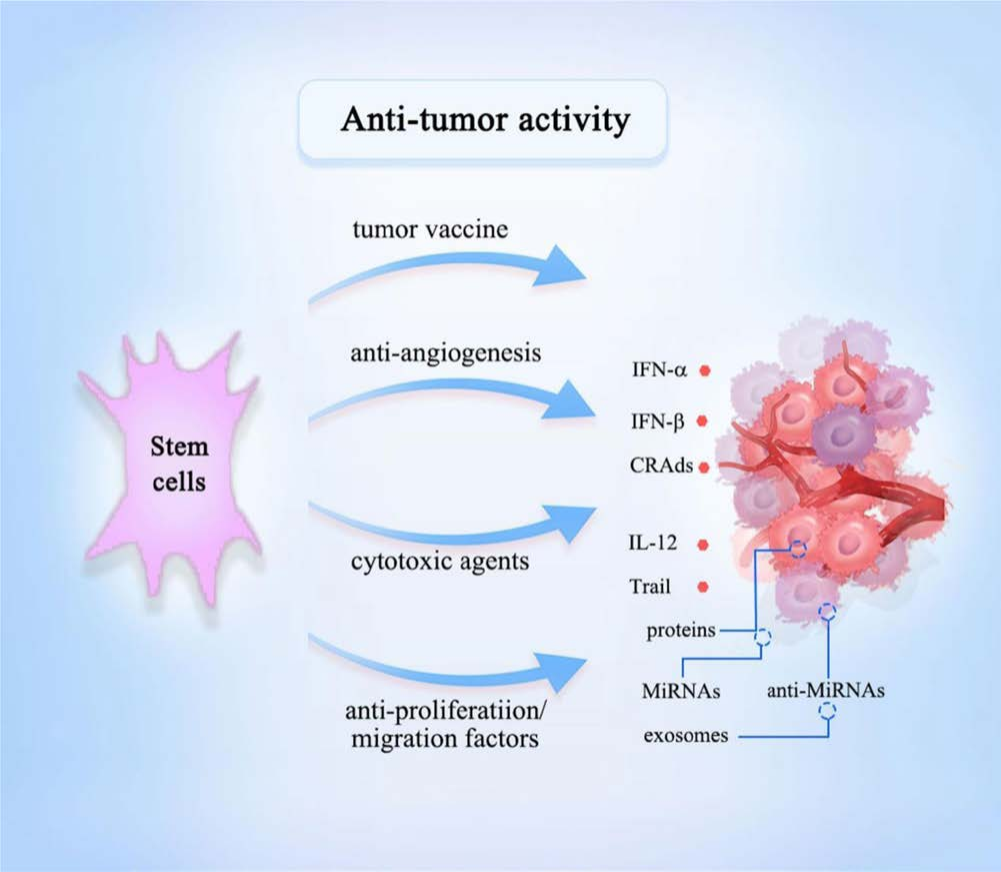
Anti-tumor activities of stem cells. Stem cells and their exosomes inhibit tumor progression through tumor vaccines, inhibition of angiogenesis, and tumor suppressors.

Exosomes released by mouse BMSCs inhibit angiogenesis in vitro and vivo targeting 4T1 cells and downregulating their expression of vascular endothelial growth factor (VEGF), furthermore, these exosomes may be associated with miR-16, which is enriched in BMSCs.^[80]^ It was shown that MSC-derived exosomes reduced the expression and secretion of VEGF by regulating mTOR/HIF-1α in breast cancer cells. Furthermore, MSC-derived exosomes inhibited angiogenesis in an in vitro angiogenesis assay, which was shown to be associated with miR-100.^[81]^ The above findings show that MSCs regulate angiogenesis to have antitumor effects against tumor cells by secreting exosomal miRNA. Exosomes from HUMSCs reduce MCF-7 and MDA-MB-231 cell migration and proliferation by upregulating miR-21-5p expression and inhibiting ZNF367 protein expression.^[82]^ miR-342-3p inhibits INHBA protein expression. Thus, BMSC-derived exosomes carrying miR-342-3p inhibit breast cancer cell proliferation and metastasis by downregulating the INHBA/IL13Rα2 signaling axis.^[83]^ In coincubation experiments with parental and cisplatin-resistant breast cancer MCF-7 and MDA-MB-231 cells, miR-1236 enriched in ADSCs was found to downregulate SLC9A1 protein expression and inactivate the Wnt/β-catenin signaling pathway. These effects increased the sensitivity of breast cancer cells to cisplatin (DDP).^[84]^ Similarly, MSC-derived exosomes as carriers of antitumor miRNAs have been employed in subsequent studies to enhance the antitumor activity of MSC-derived exosomes. Exogenous miR-379 carried by MSC-derived exosomes had a beneficial effect on breast cancer in vivo, mediated in part through the regulation of COX-2.^[85,86]^ ADSC-derived exosomes bearing miR-145 inhibited the proliferation and metastasis and promoted the apoptosis of breast cancer T47D cells.^[87]^ In breast cancer, miR-15-5p inhibits EPHA1 protein expression, and BMSC-derived exosomes harboring miR-15-5p inhibit NF-κB signaling pathway activity and EMT of breast cancer cells by suppressing EPHA1 protein expression.^[88]^ MSC-derived exosomes carrying miR-342-3p inhibit ID4 protein expression, reverse the chemotherapy resistance of breast cancer cells and inhibit tumor cell growth in vivo.^[89]^ MiR-424-5p carried by ADSC-derived exosomes promoted cytokine secretion, enhanced cytotoxicity, and reduced PD-L1 expression in MDA-MB-231 breast cancer cells. Intra-tumor administration of miR-424-5p significantly slowed tumor growth.^[90]^ MiR-3182 transported by exosomes derived from HUMSCs significantly inhibited the proliferation and migration abilities and suppressed MTOR and S6K1 expression in TNBC cells.^[91]^ Tumor dormancy renders tumor cells insensitive to therapy that is designed to target tumor cells through cell cycle inhibition. Systemic administration of MSCs loaded with antagomir-222/223 reversed tumor cell sensitivity to carboplatin-based drugs and improved host survival.^[92,93]^ Exosomes derived from MSCs carrying LNA-antimiR-142-3p were efficiently delivered to breast cancer stem cells, and they inhibited miR-142-3p and miR-150 expression and suppressed tumor stem cell clonogenicity and tumorigenicity.^[94]^ Suppression of miRNA function in tumors with MSC-derived exosomes carrying miRNA analogs, as was used in that study, is an attractive research direction. While breast cancer patients can partially benefit from radiotherapy, according to proteomics analysis, anti-inflammatory membrane-linked proteins were highly enriched in exosomes released from MSCs after chemotherapy and increased cellular sensitivity to radiation by stimulating tumor cell death.^[95]^ therefore, understanding the tumor microenvironment in breast cancer treated with neoadjuvant chemotherapy and the signaling pathways will aid in the identification of the factors related to a failure of standard treatment and improve outcomes. MSCs could be made to secrete exosomes that carry paclitaxel after coincubation with the drug, and paclitaxel released from MSC-derived exosomes showed strong tumor cell killing. In vivo, experiments showed that MSC-derived paclitaxel-carrying exosomes reduced tumor volume by 60% and inhibited tumor cell metastasis to distant organs.^[96]^ Doxorubicin encapsulated into BMSC-derived exosomes by electroporation significantly reduced breast cancer growth in a HER2/neu-overexpressing TUBO mouse model of breast cancer.^[97]^ Immortalized MSCs MSC544 cells also secrete exosomes carrying drugs, and MSC544-derived exosomes carrying paclitaxel or epirubicin exhibit significant cytotoxic effects. These cells can be used as a source for continuous mass production of drug-loaded exosomes.^[98]^ Exosomes derived from MSCs with Trail overexpression express Trail on their surface during exosome assembly. Trail-carrying MSC-derived exosomes induce caspase-related apoptotic processes in a dose-dependent manner in a variety of tumor cells and have no apparent cytotoxicity against primary human preliminary epithelial cells.^[99]^ In the MDA-MB-231 brain metastasis mouse model, Trail- and CXCR4-co-carrying exosomes and carboplatin acted synergistically to inhibit tumor brain metastasis.^[100]^ MSCs-derived exosomes also have an essential role in immunomodulation. After being transferred by ADSC-derived exosomes, miR-10a was taken up into CD4 + T cells. Upon treatment with miR-10a-loaded exosomes, the levels of RORγt and Foxp3 were increased and that of T-bet was decreased, demonstrating the potential of similar methodologies as immunotherapeutic approaches.^[101]^ In pancreatic ductal carcinoma research, BMSC-derived exosomes carrying galectin-9 siRNA and modified with surface oxaliplatin prodrugs functioned as immunogenic cell death triggers.^[102]^ MSC-derived exosome-based immunotherapy for breast cancer can ensure proper tumor targeting and can be combined with multiple approaches. In Table 3, the studies referenced in this section are summarized. A summary of factors has reported from MSCs and derivered exosomes suppress breast cancer is shown in Figure 1.

**Table 3:** The use of MSC-derived exosomes in the prevention of breast tumor development.

Exosome source	Method	Cell types	Key cargo	Effect	Proposed mechanism	References
Mouse BMSCs/MSC	In vitro and in vivo	4T1, MDA-MB- 231, MCF-7, and T47D cells	N/A	Angiogenesis↓	VEGF↓	80,81
HUMSCs	In vitro	MDA-MB- 231 and MCF-7 cells	N/A	Migration↓Proliferation↓	ZNF367↓	82
BMSCs	In vitro		MiR- 342-3p	Proliferation↓Metastasis↓	INHBA/IL13Rα2 signaling↓	83
ADSCs	In vitro	MDA-MB- 231 and MCF-7 cells	N/A	Cisplatin sensitivity↑	SLC9A1↓ Wnt/β-catenin↓	84
MSCs	In vivo	T47D and HCC- 1954 cells	MiR-379	Proliferation↓ Necrosis↓	COX-2↓	86
ADSCs	In vitro	T47D cells	MiR-145	Proliferation↓ Metastasis↓ Apoptosis↑	N/A	87
BMSCs	In vivo		MiR- 15-5p	Cell growth↓	EPHA1/NF-κB signaling↓ EMT↓	88
MSCs	In vivo	SKBR3 and MCF-7 cells	MiR- 342-3p	Chemotherapy resistance↓	ID4↓	89
ADSCs	In vitro and in vivo	MDA-MB- 231 cells	MiR- 424-5p	Cytotoxicity↑ Tumor growth↓	PD-L1↓	90
HUMSCS	In vitro	MDA-MB- 231 cells	MiR- 3182	Proliferation↓Migration↓	mTOR and S6K1 signaling↓	91
MSCs	In vitro and in vivo	MDA-MB- 231 and T47D cells	antagomir-222/223	Chemotherapy resistance↓ Survival↑	Ν/Α	93
MSCs	In vitro and in vivo		LNA-antimiR-142-3p	Clonogenicity↓Tumorigenicity↓	MiR-142-3p↓ MiR-150↓	94
MSCs	In vitro and In vivo	A549, SKOV3, and MDA- hyb1 cells	Taxol	Tumor growth↓ Cytotoxicity↑	Ν/Α	96
BMSCs	In vitro and in vivo	TUBO cells	Doxorubicin	Tumor growth↓	Ν/Α	97
MSC 544 cells	In vitro	MDA -hyb1, MDA-MB- 231, SKOV3, and A549 cells	Paclitaxel/epirubicin	Cytotoxicity↑	Ν/Α	98
MSCs	In vitro	Eleven cancer cell lines	Trail	Cytotoxicity↑	Ν/Α	99
MSCs	In vivo	MDA-MB- 231 cells	Trail/CXCR4	Tumor brain metastasis↓	Ν/Α	100
ADSCs	In vivo	CD4 + T-cells	miR-10a	T-cell immunity↑	RORγt↑ Foxp3↑ T-bet↓	101
BMSCs	In vivo	PANC -02 cells	Galectin 9 siRNA	Immunity↑ Drug accumulation↑	Galectin-9↓	102

ADSCs = adipose-derived adult stem cells, BMSCs = bone marrow mesenchymal stem cells, HUMSCs = human umbilical cord mesenchymal stem cells, MSCs = mesenchymal stem cells.

## 5. The capacity of MSCs to promote breast cancer progression

When evaluating the role of MSCs in breast cancer therapy, the possibility that MSCs also promote breast cancer should not be ignored.

**Figure 2.**
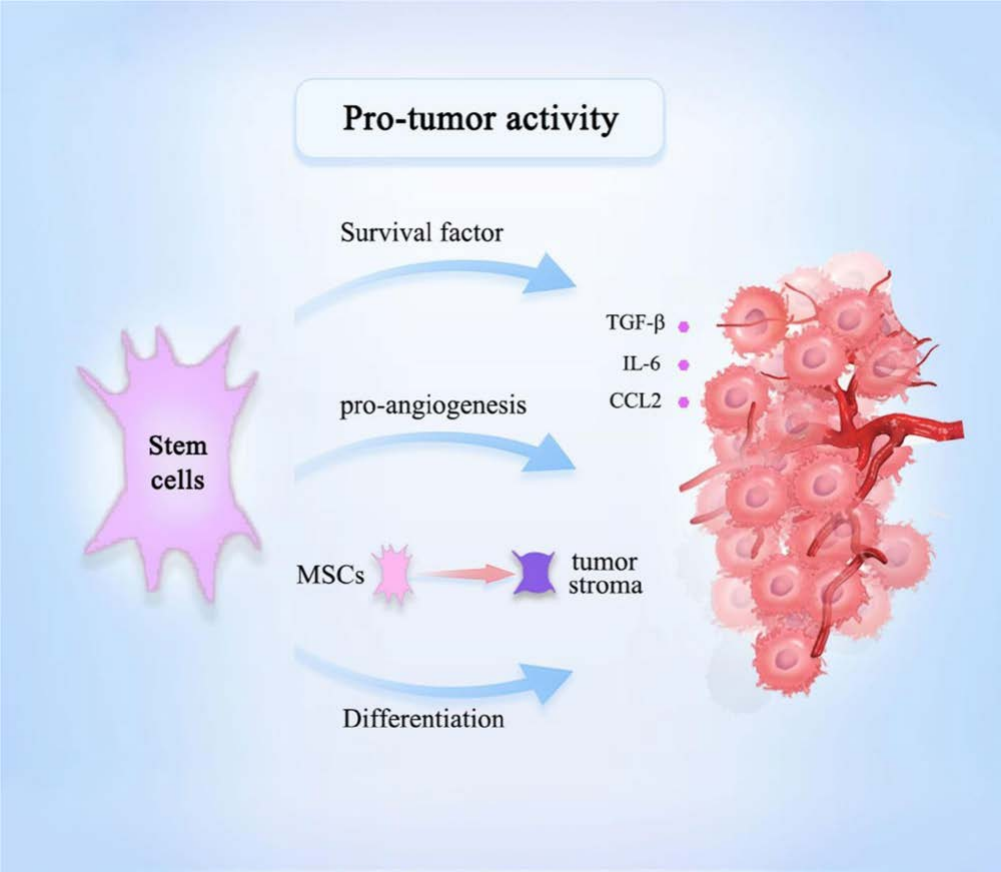
Pro-tumor activities of stem cells. Stem cells exert a promotional effect on tumors by secreting tumor survival factors, promoting angiogenesis, and differentiating into tumor stroma.

MSC-induced CD90 expression in MDA-MB-231 cells and subcutaneous injection of cocultured primary MSCs and MDA-MB-231 cells into mice resulted in an approximately 10-fold increase in tumor size and increased angiogenesis.^[103]^ Additionally, this MSC-induced increase in angiogenesis was validated in another xenograft mouse model, in which the MSCs created a vascularized environment during coculture with 4T1 cells, enhancing the colonization and proliferation of the 4T1 cells.^[104]^ Many studies have found that ADSCs increase breast cancer cell proliferation in vitro and tumorigenicity in vivo.^[105–107]^ Injection of ADSCs combined with breast cancer cells has promoted tumor cell migration and invasion in addition to tumor development in vivo.^[108,109]^ ADSCs with high CD34 levels are more likely to form xenograft tumors in nude mice, and CD34 positivity was highly correlated with angiogenesis in the model mice.^[110]^ The expression of CD34 partially accounts for the effect of ADSCs on tumor cell growth. Breast cancer cells promote their dormancy and survival in the bone marrow through microenvironmental signaling.^[111]^ The molecular mechanism by which ADSCs contribute to breast cancer cell proliferation, metastasis, and invasion may be related to the activation of the Wnt and CCL5-related signaling pathways in tumor cells.^[112,113]^ BMSCs significantly increased the proliferation and metastatic ability of tumor cells when mixed with MDA-MB-231 cells and injected into subcutaneous sites in mice, and further mechanistic studies revealed that these effects were related to the secretion of the chemokine CCL5.^[114]^ The tumor microenvironment contains a significant amount of the inflammatory cytokine TNFα, whichhas been reported to encourage BMSCs to promote tumor growth via the recruitment of monocytes/macrophages.^[115]^ Multiple cytokines/chemokines are induced in TNFα-treated BMSCs, specifically CXCR3 chemokine ligands. TNFα-treated BMSCs stimulate MDA-MB-231 breast cancer cell migration and metastasis via CXCR3 transcription.^[116]^ Furthermore, BMSCs differentiate into cancer-associated fibroblasts and become part of the microenvironment supporting tumor growth in colon carcinoma.^[117]^ In breast cancer, BMSCs in the tumor stroma may also contribute to tumor cell proliferation by differentiating into tumor-associated fibroblasts. The above findings demonstrate that BMSCs may promote breast cancer progression, and therefore, BMSCs should not be used in the treatment of breast cancer. MSC-derived exosomes containing TGF-β, C1q, and semaphorins administered as tumor treatment accelerated breast cancer progression by driving the differentiation of immature bone marrow monocyte-derived suppressor cells into highly immunosuppressive M2 macrophages.^[118]^ In addition to inducing M2-type macrophage production, MSCs reduce splenocyte, NK-cell, and CD8 + T-cell toxicity in the breast cancer microenvironment.^[119]^ Hypoxia in the tumor microenvironment induces MSCs to secrete TGF-β, which promotes tumor cell growth, migration, and invasion.^[120]^ The findings mentioned above suggest that a variety of tumor microenvironmental variables are crucial for the protumor activity of MSCs. MSCs of different stages contribute to the development of breast cancer. MSCs secrete factors, including IL-6 and CCL2, during differentiation into mature osteoblasts. Bone-derived cells promote the migration of breast cancer cells.^[121]^ The conversion of MSCs into osteoblasts in one study was induced by in vitro factors, but other factors may be present in the tumor microenvironment to induce MSC differentiation; as such, the interaction between MSCs and the in vivo tumor environment may be more complex. Table 4 presents a summary of the research referenced in this section. A summary of factors reported by MSCs promote breast cancer is shown in Figure 2.

**Table 4:** The mechanisms by which MSCs promote breast cancer development.

MSC type	Method	Cell types	Key cargo	Effect	Proposed mechanism	References
MSCs	In vivo	MDA-MB- 231 cells	N/A	Tumor growth↑ Angiogenesis↑	CD90↑	104
MSCs	In vivo	4T1 cells	N/A	Colonization↑ Proliferation↑	N/A	105
ADSCs	In vivo	MDA-MB- 231 and MCF7 cells	N/A	Proliferation↑ Migration↑ Invasion↑	Wnt and CCL5-related signaling pathways↑	113, 114
BMSCs	In vivo	MDA-MB- 231 cells	N/A	Proliferation↑ Migration↑	CCL5 signaling pathway↑	115
TNFα-treated BMSCs	In vivo	MDA-MB- 231 cells	N/A	Migration↑	CXCR3 transcription↑	117
Tumor-treated BMSCs	In vivo	4T1 cells	TGF-β C1q Semaphorins	Proliferation↑ Migration↑	M2 macrophages↑	119
MSCs	In vivo	MDA-MB- 231 and MCF7 cells	TGF-β	Migration↑ Angiogenesis↑	N/A	121
MSC- derived osteoblasts	In vivo	MDA-MB- 231, BT-474, and T47D cells	IL-6 CCL2	Migration↑	CCl2	122

ADSCs = adipose-derived adult stem cells, BMSCs = bone marrow mesenchymal stem cells, HUMSCs = human umbilical cord mesenchymal stem cells, MSCs = mesenchymal stem cells.

## 6. Conclusions

The use of stem cells in the treatment of cancer has been extensively studied, but challenges related to the immunogenicity, stability, heterogeneity, and differentiation of stem cells still need to be overcome. The risks of microvascular obstruction, tumor formation, and immunogenicity associated with stem cells limit the use of ESCs, iPSCs, and MSCs in the treatment of breast cancer. Exosomes from MSCs can be used as optimal carriers of drugs or genes to facilitate gene and/or cancer therapy due to their simple structure and tumor tropism. However, exosomes, like MSCs, can both promote and prevent the growth of breast tumors. MSC-derived exosomes have been reported to promote breast cancer development.^[122]^ Exosomes likely promote tumor cell proliferation due to their heterogeneity. For research and clinical treatment purposes, exosomes and key factors generated in ESC-CM as well as from MSCs should be further analyzed and divided into various subpopulations to clarify their functions in the treatment of breast cancer, and to develop target specific treatment of breast cancer. Further research on the communication between breast cancer cells and subtypes of exosomes derived from ES cells and MSCs may improve the knowledge of cancer biology and aid the development of delivery systems based on subpopulations of exosomes. The use of exosomes produced from stem cells in the treatment of cancer is still in the early stages. To increase the utilization of stem cells derived exosomes in clinical therapy, more research is necessary.

## Acknowledgments

I appreciate Mr. DW’s advice on this review, and hope he finds peace in paradise.

## Author contributions

**Conceptualization:** Dachun Wang.

**Investigation:** Zundong Liu.

**Writing – original draft:** Zundong Liu.

**Writing – review & editing:** Zundong Liu.
